# Successful Aortic Aneurysm Repair in a Woman with Severe von Willebrand (Type 3) Disease

**DOI:** 10.1155/2015/703803

**Published:** 2015-04-19

**Authors:** Victoria Campbell, Kevin Marriott, Rex Stanbridge, Abdul Shlebak

**Affiliations:** ^1^Department of Haematology, Imperial College Healthcare NHS Trust, London W2 1NY, UK; ^2^Department of Cardiothoracic Surgery, Imperial College Healthcare NHS Trust, London W2 1NY, UK

## Abstract

von Willebrand disease type 3 (VWD3) is a rare but the most severe form of von Willebrand disease; it is due to almost complete lack of von Willebrand factor activity (VWF:RCo). It is inherited as autosomal recessive trait; whilst heterozygote carriers have mild, or no symptoms, patients with VWD3 show severe bleeding symptoms. In the laboratory, this is characterised by undetectable VWF:Ag, VWF:RCo, and reduced levels of factor VIII < 0.02 IU/dL. The bleeding is managed with von Willebrand/FVIII factor concentrate replacement therapy. In this rare but challenging case we report on the successful excision and repair of an ascending aortic aneurysm following adequate VWF/FVIII factor concentrate replacement using Haemate-P.

## 1. Introduction

von Willebrand disease (VWD) is a bleeding disorder due to defective von Willebrand factor (VWF:Ag) [1–3]. VWF is a large and complex plasma glycoprotein which is essential for normal haemostasis by mediating platelet adhesion and promoting platelet aggregation to subendothelial tissues in the vessel wall following injury. It also binds and protects factor VIII from proteolysis in the plasma, prolonging its half-life [4, 5]. VWF circulates in a multimeric form of up to 20,000 kDa linked by disulphide-linked dimers, with the largest multimers having the highest haemostatic function [6, 7]. The current classification of VWD (Table 1) consists of six distinct types [1]. Whilst types 1 and 3 are the result of quantitative VWF deficiency, the four type 2 subclasses are the results of a qualitative defect [1, 3]. The commoner type 1 (60–70% of cases) has several subtypes including probable, definite, and severe type 1. Type 2 is subclassified into 2A, 2B, 2M, and 2N depending on VWF:RCo/VWF:Ag ratio (<0.6 in 2A, 2B, and 2M but <0.7 in type 2N), ristocetin-induced platelet aggregation (RIPA), and multimeric patterns. Type 2A includes 2C, 2D, and 2E previously known forms [1, 3, 7]. Type 1 and most of type 2 cases are inherited as autosomal dominant [1, 3] whilst type 3 is inherited as autosomal recessive. VWD3 is characterised by undetectable VWF:Ag and reduced FVIII < 0.02 IU/dL [8]. It is rare and affects 0.1–5.3 individuals per million [9–11] with heterozygotes being asymptomatic or having mild bleeding phenotype [2, 12, 13]. The incidence in Europe and North America is comparable (1.51 and 1.38 per million, resp.). It is highest among Arab Community due to consanguinity. The laboratory diagnosis of the severe form VWD3 can be made more readily than other forms due to the very low levels of both VWF:Ag and FVIII reflecting the clinical severity. The hallmark of VWD3 is markedly prolonged bleeding time reflecting the extremely low VWF:Ag in plasma and platelets but VWD3 patients have normal platelet count [14].

The objective of replacement therapy using VWF/FVIII factor concentrate is to prevent bleeding during surgery (prophylaxis for surgery) by raising VWF:RCo (to correct primary haemostasis) and FVIII to normal levels (to correct the coagulation function for secure longer term haemostasis). The minimum criteria for such a concentrate are as follows: (i) the concentrate must contain enough biologically active VWF:Ag to correct the primary haemostasis defect and to stabilise the normally produced endogenous FVIII; (ii) it should be virally inactivated; and (iii) its efficacy has been validated in clinical studies with supporting pharmacokinetic data. Only few VWF/FVIII concentrates [15–23] available meet the above criteria (Table 2). Because endogenous FVIII production is normal, continued administration of large amounts of FVIII may result in an undesirably high plasma FVIII level. The dosing schedule and the duration of VWF/FVIII concentrate replacement therapy (Table 3) vary according to the procedure and the urgency [24, 25]. Thompson et al. [26] recommended maintaining a trough VWF:RCo of around 1.0 IU/dL using a twice daily protocol for first 72 hours and then to aim for >0.5 IU/dL thereafter for 7–10 days; furthermore, the aim is to keep FVIII <2.0 IU/dL in view of associated risk of venous thrombosis. The challenge is to titrate the dose and frequency to the patient response to the concentrate of choice; this was a major challenge in our case, an overseas patient with no previous factor VWF:VIII concentrate recovery data available. In the postoperative period, recent studies have shown efficacy by monitoring and maintaining VWF:RCo >0.5 IU/dL for 6 days [27–29]. Maintaining FVIII >0.5 IU/dL for 7–10 days using a suitable VWF:RCo concentrate has also proved an effective alternative and was common practice before rapid turn-around-time VWF:RCo measurements became available [30].

## 2. Case Presentation

We report on a 32-year-old female of North-African origin with type 3 von Willebrand disease referred to our cardiothoracic surgical centre for elective surgery. She presents with an 18-month history of chest pain and breathlessness on minimal exertion. Cardiac evaluation had revealed an ascending aortic aneurysm measuring 4.8 cm × 4.3 cm but further interval follow-up imaging showed the aneurysm to be expanding at 5.2 cm × 4.8 cm.

**Figure 1:**
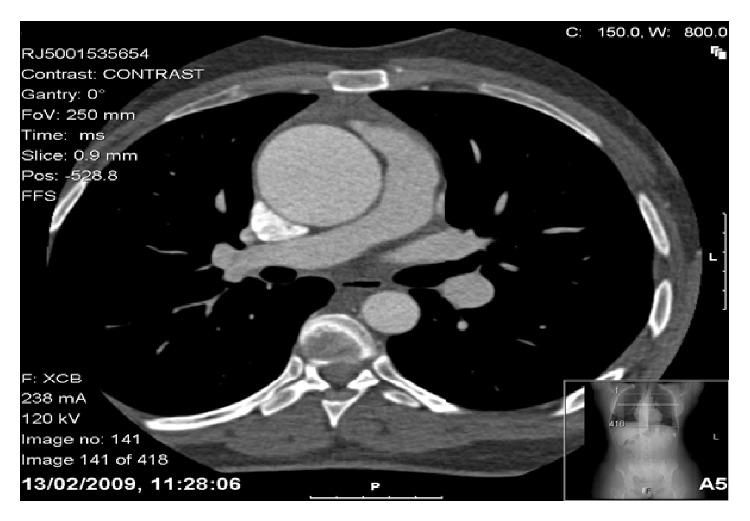
Contrast CT image showing large ascending aortic aneurysm measuring 6.3 × 5.4 cm.

She had a life-long severe bleeding phenotype with extensive ecchymosis, epistaxis, and gum bleeding necessitating hospitalisation on countless occasions from infancy. Since menarche, at 12 years of age, she has had menorrhagia leading to severe iron deficiency anaemia requiring iron supplementation, repeated red cell transfusions, and on-demand VWF:FVIII factor concentrate including Wilfactin (high-purity plasma derived von Willebrand factor concentrate). Aged 14 a minor road traffic accident resulted in catastrophic internal bleeding requiring transfusion of 27 units of red cells. At the age of 18 years following rupture of an ovarian cyst she had a massive haemorrhage requiring 48 units of red cells; a second ovarian cyst rupture resulted in recorded haemoglobin of 2.0 g/dL. She had received plasma products ranging from fresh frozen plasma (FFP) and cryoprecipitate to VWF:VIII factor concentrate when available, contracting hepatitis C as a result of treatment. There is a strong family history, two siblings with severe bleeding phenotypes: one dying aged 8 years from prolonged epistaxis and the other requiring a 30-unit red cell following circumcision. Three other siblings have no bleeding symptoms. Parents are asymptomatic but consanguineous. Baseline laboratory investigations (Table 4) including full blood count showed a haemoglobin of 9.6 g/dL and a microcytic hypochromic picture with a normal platelet count at 346 × 10^9^/L and normal WBC at 4.5 × 10^9^/L. Her coagulation screen showed a normal prothrombin time (PT) of 10.5 sec (9.4–11.3), prolonged activated partial thromboplastin time (APTT), 51.0 sec (25.0–30.7), and a normal thrombin time (TT), 14.3 sec (12.9–15.2). The 50 : 50 mix showed an APTT of 28 sec (23.0–29.0). FVIII was 0.09 IU/dL (0.45–1.50) and VWF:Ag 0.03 IU/dL (0.40–2.40) with undetectable ristocetin cofactor (VWF:RCo) and undetectable collagen binding confirming type 3 von Willebrand disease. She had no inhibitor. The ferritin was depleted at 3 *μ*g/L (20–300). Her blood group was A Rh D positive. Hepatitis B surface Ag was negative with a positive anti-HBc and anti-HBs of 10.11 miu/mL. Hepatitis C antibody was positive, type 1b genotype. An ascending aortic aneurysm was confirmed on CT scan measuring 6.3 × 5.4 cm (Figure 1).

**Figure 2:**
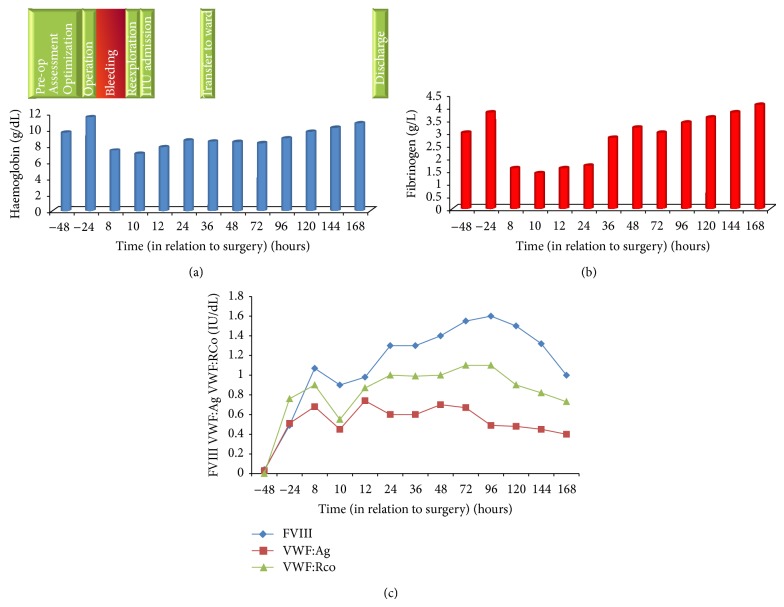
The clinical progress of the patient and the laboratory parameters throughout (time in hours in relation to surgery). The laboratory parameters include (a) haemoglobin g/dL, (b) fibrinogen g/L, and (c) factor assays for FVIII, VWF:Ag, and RCo in IU/dL.

**Figure 3:**
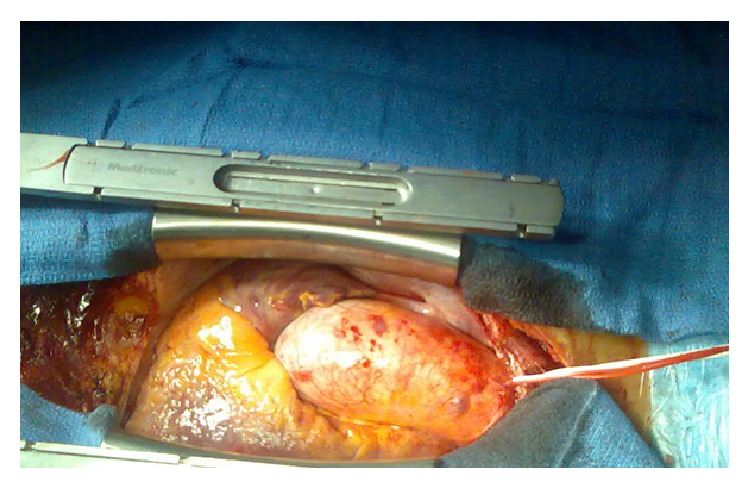
Intraoperative clinical image showing the pale blue aneurysm with very thin wall and features of localised imminent rupture potential.

Figure 2 shows the preoperative, intraoperative, and postoperative clinical course and haemostatic laboratory parameters throughout the procedure. She was admitted 48 hours preoperatively for assessment including Haemate-P infusion to determine her response at a dose of VWF:RCo 80 U/kg. There was a good response in her VIII from 0.09 to 0.49 IU/dL, a rise in VWF:Ag from 0.03 to 0.51 IU/dL, and VWF:RCo from 0.0 to 0.76 IU/dL (Table 4). Surgery was therefore planned with Haemate-P (100 U/kg) being administered shortly before surgery to ensure adequate response in addition to tranexamic acid 1 g 8-hourly by intravenous route. A 5-cm pale blue aneurysm was identified during surgery; it has very thin wall and localised imminent rupture potential (Figure 3); the excision and repair were uneventful with a bypass time of 56 minutes and a minimal blood loss. Eight hours postoperatively, she became hemodynamically unstable with hypotension and tachycardia despite red cell and platelet transfusion, with an estimated blood loss of 500 mL (Figure 3). Her haemoglobin had fallen to 7.4 g/dL (pre-op 11.5 g/dL) and platelet count 130 × 10^9^/L, PT 13.1 sec, APTT 30.8 sec, TT 17.1 sec, and fibrinogen 1.6 g/L (Table 4). An echocardiogram showed a large pericardial effusion (1.8 cm) around the right atrium and ventricle causing compression with threatening tamponade. Surgical reexploration found considerable clot burden with low grade bleeding at the angle of aortotomy and around the sternal wires. Closure was performed after irrigation with haemostasis secured. Postoperatively, she was monitored on ITU for 24 hours before transfer to the ward. Recovery thereafter was uneventful with discharge 8 days postoperatively; Haemate-P was reduced to once daily 72 hours postoperatively. Our patient received thromboprophylaxis with enoxaparin 40 mg S/C daily from day 3 till discharge. Histological examination of the excised aneurysm showed focal thinning of the media with loss of elastin, fibrosis and local myxoid change, mild fibrointimal proliferation, and focal nonspecific medial and adventitial perivascular chronic inflammation. Further immunological investigations confirmed negative syphilis, negative anti-unclear antibody, and negative rheumatoid factor but high level of background fluorescence staining for ANCA. The myeloperoxidase antibody was very high at 115.0 EliA U/mL (0.0–7.0) and very high proteinase 3 Ab at 144.0 EliA U/mL (0.0–7.0). Complement C3 and C4 were normal.

To our knowledge, there are no similar cases of aortic aneurysm repair in a patient with VWD3. We aim to use this rare case to highlight the complexity of managing patients with a severe bleeding diathesis undergoing high risk procedures and highlight the importance of performing such surgery in a specialised centre in managing such patients with access to specialist laboratory to allow timely laboratory monitoring around the clock. The importance of an agreed and documented plan with multidisciplinary input from haematology, cardiothoracic surgery, anaesthetics, and the laboratory including the blood transfusion department is paramount. We review the recommendations on dosing, monitoring, and duration of therapy for surgical prophylaxis with particular emphasis on patients with VWD3.

## 3. Discussion

We report on a successful excision and repair of an ascending aortic aneurysm on bypass in a woman with a severe form of VWD. This is a high risk procedure at best times, including hemostatically competent candidates, and is associated with significant morbidity and mortality in particular risk of bleeding requiring major transfusion support with the need for massive red cell and other blood products transfusion. Due to complexity and life-threatening nature of the procedure we adopted an aggressive dosing schedule similar to that adopted by Thompson et al. [26]. We want to stress on the importance of multidisciplinary approach and a specialist laboratory to ensure the timely monitoring of VWF:RCo and FVIII levels to enable optimal replacement therapy. The case has presented us with several challenges including lack of data on previous haemostatic pharmacokinetic response to VWF/FVIII concentrate being an overseas visitor who was treated on demand with different VWF:FVIII concentrates over the years most recently with Wilfactin for spontaneous bleeding; she was moderately anaemic and is hepatitis C antibody positive. Additional potential risk is the contemplated platelet dysfunction due to the bypass circuit. Accordingly, the patient was admitted 48 hours preoperatively to enable haemostatic assessment.

Haemate-P was used being the VWF:FVIII factor concentrate we are most familiar with in our centre and in the UK. It has excellent safety record over spanning nearly 40 years of clinical use in regard to the blood borne infections. Haemate-P (Humate-P) and Alphanate are most widely used in Europe and USA, respectively; both are lyophilized concentrate of purified VWF and FVIII, and they also contain fibrinogen and albumin. Due to their processing, the quantity of high-molecular-weight multimers (HMWM), the most hemostatically active moiety of VWF, is decreased compared to fresh plasma with Haemate-P containing the highest levels. Furthermore, the ratio of VWF:RCo to FVIII content differs between the two concentrates with highest ratio in Haemate-P ranging from 2.7 to 1.6, compared to a ratio ranging from 1.2 to 0.5 for Alphanate. These characteristics explain their significantly different dosing schedule and frequency of administration and therefore are not interchangeable. Adverse events (AEs) related to VWF/FVIII concentrates are rare but variable, ranging from urticaria, skin rashes, pruritus, oedema, phlebitis, and chest tightness to severe anaphylaxis. In these situations the infusions must be stopped immediately and appropriate resuscitation measures instigated. The efficacy and tolerability of Haemate-P are well documented in several prospective and retrospective studies. In one of the largest retrospective studies involving 100 patients with VWD including 37 with VWD3, the responses rated excellent or good in 97% and 95% among patients undergoing surgery or treated for bleeding, respectively [27]. In a large retrospective Canadian study involving 97 patients including 28 VWD3, the efficacy was rated as excellent in 99% of invasive procedures, in 97% of bleeding episodes, and in all prophylactic treatments [30, 36]. Tolerability was good, with no serious related AEs. Dobrkovska et al. [30] in their study on 26 VWD3 patients reported that patients undergoing major surgery required higher dosage and for longer periods as compared with minor surgery [28]. Haemate-P was further assessed by two USA prospective open label nonrandomised studies in bleeding patients and in patients undergoing surgery [26, 29]. In bleeding patients the response was deemed as excellent in 98% with a median loading dose and daily maintenance dose at 67.0 VWF:RCo U/kg and 74.0 VWF:RCo U/kg, respectively. Thompson et al. [26] reported efficacy as excellent or good efficacy on all evaluable patients and with good tolerability at a dose of 82.3 VWF:RCo U/kg and 52.8 VWF:RCo U/kg for loading and daily maintenance, respectively. The two studies reported 9 AEs (including vasodilation, 2 events of paraesthesia, allergic reaction, pruritus, peripheral oedema, pseudothrombocytopenia, and extremity pain). In another open label multinational study, 91% of patients who underwent surgery responded excellently [15]. Finally, Haemate-P was studied in a prospective study of 29 patients with 9 classified as VWD3 undergoing surgery with response rated as excellent or good in 96.3% on the day of surgery and 100% next day [37].

Alternative VWF:FVIII concentrates are available in Europe and North America for use in routine clinical practice including Fanhdi (Grifols), Wilate (Octapharma), Wilfactin (LFB), and Biostate (CSL Behring) (Table 2). Although these concentrates have different pharmacokinetics to Haemate-P and Alphanate, they have been found to have very similar clinical efficacy in prospective and retrospective studies [15, 18, 21, 22, 31–35, 38–40]. The recommended doses of VWF/VIII concentrates (Table 3) depend on the product used and the clinical circumstance of the patient [24–26]. Critically, the VWF:VIII ratio is important to ensure that VWF is raised to an appropriate level whilst maintaining a concentration of factor VIII that will not induce thrombosis. When comparing VWF:VIII factor concentrate with high ratio (Haemate-P) or low ratio (Wilate) in an animal model, a higher cumulative exposure effect with Wilate by 84% was noted, suggesting that these high FVIII levels could be avoided using a concentrate with high ratio [41]; however, this has not been a problem in clinical practice in VWD patients with no accumulation of FVIII or VWF and no thromboembolic events included in the recently reported large experience in children following the administration of 260,000 IU Wilate [31]. Other reports have confirmed similar safety profile and no adverse events related to cumulative exposure to Wilate [22, 32, 40]. Factor concentrate with higher ratios is expected to be favourable for clinical efficacy as the HMWM are essential for platelet adhesion to sites of vascular injury [3]. Comparison of different concentrates yields a uniform VWF:RCo recovery of 0.021–0.024 IU/dL per U/kg [42] but reveals significant differences in specific activity (function: antigen) and in their content of HMWM [43, 44]. In practice, all the commonly used VWF/VIII concentrates can be considered effective and well tolerated for prevention or treatment of bleeding despite the differences in purity and viral inactivation steps. Tranexamic acid is a useful adjunctive agent. When bleeding persists despite apparently normal plasma levels of VWF:RCo, platelet transfusion may be helpful.

VWF:FVIII concentrates should be used with caution in patients who have known risk factors for thrombosis; they are rarely linked with venous thromboembolism (VTE) associated with high levels of FVIII. VTE risk factors include previous thrombosis, obesity, surgery, immobility, oestrogen contraceptive pill, hormone replacement therapy (HRT), thrombophilia, antiphospholipid syndrome, cancer, and use of antifibrinolysis therapy. Some of these risk factors were applicable to our patient including major surgery, high FVIII, and the use of antifibrinolytic agents and therefore FVIII was monitored on daily basis and LMWH thromboprophylaxis was employed appropriately.

## Figures and Tables

**Table 1:** Classification of VWD (adapted from Laffan et al. [3]).

Type	Nature of VWF defect	Phenotypic changes	Mode of inheritance
1	*Partial quantitative deficiency *	(i) Includes rapid VWF clearance (e.g., VWF Vicenza) mutations (ii) Requires VWF:RCo/VWF:Ag ratio >0.6	(i) Predominately autosomal dominant inheritance when VWF <0.3 IU/dL (ii) VWF mutations with levels >0.3 IU/dL show variable penetrance
2	*Qualitative defects *		
2A	Decreased VWF-dependent platelet adhesion with selective deficiency of HMWM	VWF:RCo/VWF:Ag ratio <0.6	(i) Mostly autosomal dominant
2B	Increased affinity for platelet GPIb	(i) VWF:RCo/VWF:Ag ratio <0.6 (ii) Typically associated with thrombocytopenia (iii) Should be distinguished from PT-VWD, by further testing by platelet agglutination tests or genetic testing (iv) Cases with normal VWF multimer and platelet count have been described	(i) Autosomal dominant
2M	Decreased VWF-dependent platelet adhesion without selective deficiency of HMWM	(i) This also includes defects of VWF collagen binding	(i) Autosomal dominant
2N	Markedly decreased binding affinity for FVIII	(i) VWF:RCo/VWF:Ag ratio <0.7 (ii) Should be distinguished from mild haemophilia A	(i) VWF:FVIII binding defects are more commonly due to compound heterozygote with a VWF null allele rather than the classical homozygous state
3	*Virtually complete deficiency *	(i) Equivalent to <0.03 IU/dL in most assays (ii) Bleeding symptoms in up to 48% of obligate carriers	(i) Autosomal recessive, frequent null VWF alleles

HMWM = high molecular weight multimers, PT-VWD = platelet type VWD.

**Table 2:** VWF:FVIII concentrates validated in clinical studies involving relatively large number of VWD patients.

Concentrate	Manufacturer	VWF:RCo/FVIII^∗^	Viral inactivation	Purification methodology
Alphanate [15]	Grifols (USA)	1.2	Solvent detergent, dry heat	Heparin ligand chromatography,
Biostate [17, 31, 32]	CSL Behring	2.0	Solvent detergent, dry heat	precipitation, and heparin ligand chromatography
Fanhdi [15, 33]	Grifols (SP)	1.6	Solvent detergent, dry heat	Precipitation, heparin ligand chromatography
Haemate-P (Humate-P) [16, 19, 20, 26, 27]	CSL Behring	2.5	Pasteurization	Polyelectrolyte precipitation
Wilate [34]	Octapharma	0.8	Solvent detergent, dry heat	Affinity chromatography, size exclusion
Wilfactin [18, 35]	LFB (Lille)	60	Solvent detergent, nanofiltration, and dry heat	Ion-exchange, affinity chromatography

^∗^Ratio of ristocetin cofactor activity (VWF:RCo) to FVIII activity expressed as IU/mL.

**Table 3:** Recommended factor VWF/FVIII concentrates dosages for different indications in the treatment of VWD3 [24–26].

Indication	Dose VWF:RCo units	Frequency of infusions	Duration of treatment	Target VWF:RCO	Target FVIII
Major surgery		Every 8–24 hours	7–14 days	Maintain trough >0.5 IU/dL	Maintain trough >0.5 IU/dL
Loading	40–60 U/kg				
Maintenance	20–40 U/kg				
Minor surgery		Every 12–48 hours	1–5 days	Maintain trough >0.5 IU/dL	Maintain trough >0.5 IU/dL
Loading	30–60 U/kg				
Maintenance	20–40 U/kg				
Tooth extraction	20–40 U/kg	Single infusion	Single infusion	Achieve trough >0.5 IU/dL	Achieve trough >0.5 IU/dL
Spontaneous or traumatic bleeding	25 U/kg	Daily infusion	2–4 days	Maintain trough >0.5 IU/dL	Maintain trough >0.5 IU/dL

**Table 4:** Baseline and the immediate perioperative laboratory results.

Investigation	Reference range	Baseline	Preoperative assessment following optimisation	8 hours postoperatively
Haemoglobin	11.5–15.1 g/dL	9.6	11.5 (following 2-unit packed red cell transfusion)	7.4
Platelet count	147–397 ×10^9^/L	346	318	130
Ferritin	20–300 ug/L	3.0		
Prothrombin time (PT)	9.4–11.3 sec	10.5	10.9	13.1
Activated partial thromboplastin time (APTT)	25.0–30.7 sec	51.0	27.8	30.8
Thrombin time (TT)	12.9–15.2 sec	14.3	14.9	17.1
Fibrinogen	1.8–4.1 g/L	3.0	3.7	1.6
50 : 50 mixing with normal plasma	23.0–29.0 sec	25.0	28.0	29.0
Factor VIII (FVIII)	0.45–1.50 IU/dL	0.09	0.49^∗^	1.07
von Willebrand factor (VWF:Ag)	0.40–2.40 IU/dL	0.03	0.51^∗^	0.68
Ristocetin cofactor (RCo)	0.40–2.40 IU/dL	0.00	0.76^∗^	0.90

^∗^Following a Haemate-P infusion at a dose of VWF:RCo 80 U/kg.
